# Constitutively Active MAVS Inhibits HIV-1 Replication via Type I Interferon Secretion and Induction of HIV-1 Restriction Factors

**DOI:** 10.1371/journal.pone.0148929

**Published:** 2016-02-05

**Authors:** Sachin Gupta, James M. Termini, Biju Issac, Elizabeth Guirado, Geoffrey W. Stone

**Affiliations:** 1 Department of Microbiology and Immunology, Miami Center for AIDS Research and Sylvester Comprehensive Cancer Center, University of Miami Miller School of Medicine, Miami, Florida, United States of America; 2 Division of Bioinformatics, Biostatistics and Bioinformatics Core, Sylvester Comprehensive Cancer Center, University of Miami Miller School of Medicine, Miami, Florida, United States of America; Lady Davis Institute for Medical Research, CANADA

## Abstract

Type I interferon is known to inhibit HIV-1 replication through the induction of interferon stimulated genes (ISG), including a number of HIV-1 restriction factors. To better understand interferon-mediated HIV-1 restriction, we constructed a constitutively active form of the RIG-I adapter protein MAVS. Constitutive MAVS was generated by fusion of full length MAVS to a truncated form of the Epstein Barr virus protein LMP1 (ΔLMP1). Supernatant from ΔLMP1-MAVS-transfected 293T cells contained high levels of type I interferons and inhibited HIV replication in both TZM-bl and primary human CD4+ T cells. Supernatant from ΔLMP1-MAVS-transfected 293T cells also inhibited replication of VSV-G pseudotyped single cycle SIV in TZM-bl cells, suggesting restriction was post-entry and common to both HIV and SIV. Gene array analysis of ΔLMP1-MAVS-transfected 293T cells and trans-activated CD4+ T cells showed significant upregulation of ISG, including previously characterized HIV restriction factors Viperin, Tetherin, MxB, and ISG56. Interferon blockade studies implicated interferon-beta in this response. In addition to direct viral inhibition, ΔLMP1-MAVS markedly enhanced secretion of IFN-β and IL-12p70 by dendritic cells and the activation and maturation of dendritic cells. Based on this immunostimulatory activity, an adenoviral vector (Ad5) expressing ΔLMP1-MAVS was tested as a molecular adjuvant in an HIV vaccine mouse model. Ad5-Gag antigen combined with Ad5-ΔLMP1-MAVS enhanced control of vaccinia-gag replication in a mouse challenge model, with 4/5 animals showing undetectable virus following challenge. Overall, ΔLMP1-MAVS is a promising reagent to inhibit HIV-1 replication in infected tissues and enhance vaccine-mediated immune responses, while avoiding toxicity associated with systemic type I interferon administration.

## Introduction

Type I interferons are key mediators of both innate and adaptive immune responses [1–3], including inhibition of viral replication [4,5]. Currently, type I interferons are used in the treatment of hepatitis C and hepatitis B viral infections [6–8]. Type I interferons can also inhibit HIV-1 replication [9–11], blocking early steps in the HIV-1 viral life cycle [12]. While type I interferon has been tested as an anti-HIV therapy in clinical trials [13,14], side effects preclude its use compared to the current generation of anti-retroviral drugs. Interferon-mediated inhibition of HIV-1 has been associated with the upregulation of a number of interferon stimulated genes (ISG) [15–17]. These studies have led to the identification of a number of interferon-induced ISG that restrict HIV-1 replication, including Tetherin, TRIM5α, and Viperin [18–20]. ISG induction and HIV-1 infection has been evaluated both in culture cells and primary host cells, including CD4+ T cells and macrophages [21].

MAVS (also called IPS-1 or VISA) acts as an adapter protein for the pattern recognition receptor (PRR) molecules RIG-I and MDA-5. As such, MAVS is a key mediator of antiviral immunity following RIG-I/MDA-5 sensing of viral RNA [22–24]. MAVS activation induces type I interferon through transcription factors IRF3, IRF7, and NF-κB [25,26]. MAVS signaling is known to play a role in control of number of viral infections, including dengue and hepatitis C, through the induction of type I interferons [27–29]. MAVS has also been implicated in the induction of adaptive immunity via increased type I interferon expression [23,30]. Importantly, MAVS activation requires the complexation of MAVS with RNA-bound RIG-I or MDA-5 [26]. This complex forms large tubular structures on the mitochondrial surface, leading to interferon induction via TBK-1 mediated phosphorylation of IRF-3 and IRF-7 and FADD mediated activation of NF-κB [23,31].

Despite the central role of MAVS in viral RNA-mediated interferon induction and innate and adaptive immune responses, MAVS stimulation has not been studied in the context of HIV-1 infection. Furthermore, ISG induction in primary targets of HIV-1 infection, such as human CD4+ T cells, has not been evaluated. For example, it is unclear whether soluble factors generated by constitutive MAVS signaling, other than IFN-α/β, may contribute to suppression of HIV-1 replication.

In order to generate a constitutively active MAVS, we fused full length MAVS to the transmembrane domain of the Epstein Barr viral protein LMP1. LMP1 is known to self-aggregate via its transmembrane domain [32,33]. For example, fusion of the LMP1 transmembrane domain to the intracellular domain of the molecule CD40 led to constitutive CD40 signaling, even at low protein concentrations [34]. We hypothesized that fusing MAVS to the LMP1 transmembrane domain (generating ΔLMP1-MAVS) would lead to the spontaneous aggregation of MAVS and constitutive MAVS signaling.

In this study we evaluated inhibition of HIV-1 replication in TZM-bl culture cells and primary human CD4+ T cells following incubation with supernatant from ΔLMP1-MAVS transfected HEK-293T cells. Depletion studies suggested inhibition was mediated by type I interferon, in particular IFN-β. HIV-1 inhibition was accompanied by the upregulation of a number of ISG associated with HIV-1 restriction including Viperin, Tetherin, MxB, and ISG56 [18,19,35–37]. Finally, dendritic cells were activated following ΔLMP1-MAVS transduction and we observed enhanced adaptive immune responses when ΔLMP1-MAVS was used as a vaccine molecular adjuvant. Together, these data support a model where targeted delivery of constitutively active MAVS to sites of HIV-1 replication leads to the local secretion of high levels of type I interferon, inhibiting HIV-1 replication and enhancing anti-HIV-1 adaptive immune responses.

## Materials and Methods

### Ethics Statement

All animal experiments were performed in accordance with national and institutional guidance for animal care and were approved by the University of Miami Institutional Animal Care and Use Committee (IACUC), assurance number A3224-01, protocol number 14–011. This study was carried out in strict accordance with the recommendations in the Guide for the Care and Use of Laboratory Animals of the National Institutes of Health. Animals were monitored daily, and all animals remained healthy throughout mouse experiments outlined in this manuscript. Mice were euthanized by carbon dioxide inhalation for all experiments outlined.

### Cells, Viruses and Reagents

293T cells (American Type Culture Collection, Manassas, VA) were cultured in complete DMEM medium (Dulbecco’s modified Eagle medium (DMEM) supplemented with 10% fetal bovine serum (FBS) (Hyclone Inc.), 2 mM L-glutamine, 100 U/ml penicillin and 100 μg/ml streptomycin). TZM-bl cells were obtained through the National Institutes of Health AIDS Research and Reference Reagent Program (contributed by John C. Kappes and Xiaoyun Wu) and were cultured in complete DMEM medium. HIV-1 virus stocks were prepared from infectious full-length HIV-1 Bal clone by transfection of 293T cells as described in Gupta et al. [38]. VSV-G trans-complemented single-cycle SIV was produced by co-transfection of 293T cells with the Gag-Pol expression construct pGPfusion, 5 μg of an expression construct for the Indiana or the New Jersey serotype of VSV-G and a full-length proviral DNA construct for each scSIV strain as previously described [39]. RosetteSep Human CD4+ T Cell Enrichment Kit was purchased from StemCell Technologies (Vancouver, BC, Canada).

### Construction and preparation of DNA plasmids

To construct LMP1 plasmid, cDNA was prepared from Raji B cells (American Type Culture Collection, Manassas, VA) and the LMP1 sequence of EBV was isolated by polymerase chain reaction and found to be identical to the reference sequence (GenBank M58153.1) as described in Gupta et al [38]. ΔLMP1-MAVS was constructed by fusing DNA encoding the N-terminal 190 amino acids of LMP1 (beginning with the ATG start codon) with the full-length murine MAVS gene (amino acids 1–504), followed by a TGA translational stop codon. All plasmids were propagated in *Escherichia coli* strain TOP10. Endotoxin-free DNA plasmid preparations were prepared using an Endofree Giga plasmid kit (Qiagen). Plasmids were further purified to remove residual endotoxins with additional Triton-X114 extractions as previously described [40]. Plasmid endotoxin level was <0.2 EU/ml for all constructs as confirmed by LAL endotoxin assay (Lonza Inc.).

### Transient transfection and Western blotting of protein constructs

293T cells were transiently transfected with plasmid constructs using Genjet-plus Transfection Reagent (Signagen Laboratories, Iamsville, MD). Forty-eight hours later, supernatants were centrifuged and filtered with a 0.45 μm filter to remove debris. Filtered supernatant was reduced with 2-mercaptoethanol, loaded onto sodium-dodecyl sulfate–10% polyacrylamide gels (10% SDS-PAGE) (BioRad), electrophoresed, and blotted onto PVDF membranes (Pierce). The membranes were blocked using 5% (w/v) dry milk and then probed with primary antibodies using a 1:1,000 dilution of MAVS antibody specific to carboxyl terminal domain (clone V-17, Santa Cruz Biotechnology, Santa Cruz, CA). Following incubation in the secondary antibody, the membranes were washed and then incubated in HRP substrate (Pico chemiluminescence; Pierce). Membranes were placed on Whatman 3MM filter paper and exposed to film (BioMax; Kodak, Rochester, NY).

### Luciferase reporter assay

NF-κB and IFN-β induction were measured using a dual-luciferase reporter assay system (Promega) according to manufacturer protocol. 293T cells seeded on 24-well plates were transiently transfected with 30ng of either NF-κB or IFN-β firefly luciferase reporter plasmid together with 4 ng pRL-TK and 300ng of LMP1 or ΔLMP1-MAVS plasmids or empty control plasmids. As positive controls, pcDNA3.1-FLAG-TRAF6 and pcDNA3.1-ΔRIG-I were used for NF-κB and IFN-β respectively. Luciferase activity was measured in the total cell lysate after 36-48h.

### NF-κB in vitro activity assay

A NF-κB reporter cell line (293-SEAP) was generated by Dr. Richard Kornbluth and provided as a gift to monitor NF-κB activation by LMP1. The 293-SEAP cell line was derived from HEK293 cells (ATCC) and contains the gene for secreted embryonic alkaline phosphatase (SEAP) under the control of an NF-κB regulated promoter [41]. 80,000 293-SEAP reporter cells, grown in DMEM medium with 10% FBS, were plated in each well of a 96-well plate. Viral stocks of Ad5-LMP1 or Ad5-ΔLMP1-MAVS were serially diluted and added to the reporter cells in triplicate, in the presence or absence of 1ug/ml doxycycline (Alfa Aesar). After 36 h, 10 μl/well of the supernatants was added to the wells of a 96-well assay plate together with 100 μl/well of QUANTI-Blue Alkaline Phosphatase substrate (InvivoGen). Plates were incubated for 20 min at 20°C and OD was read at 650 nm.

### TZM-bl Virus Inhibition Assay

293T cells were plated at 300,000 cells per well in 24-well plate and next day transfected with pcDNA3.1 plasmids encoding GFP, LMP1, or ΔLMP1-MAVS using Genjet plus transfection Reagent (Signagen Laboratories, Iamsville, MD) in serum-free medium. All assays were performed in triplicate. Twenty-four hours following transfection, plates were rinsed twice and cultured in DMEM media supplemented with 10% FBS. Twenty-four hours later, the cell culture supernatant was collected, clarified by centrifugation at 500 x g for 10 min, and filtered through a 0.45μm-pore-size membrane (Millipore, Bedford, MA). TZM-bl indicator cells were plated as 250,000/well of 24 well plate in 500ul of complete DMEM media containing 10% FBS, L-glutamine, penicillin, and streptomycin. For the direct experiments, 500ul of 293T cell culture supernatants were added to the TZM-bl indicator cell and incubated for 24 hrs at 37°C. For transwell separation of 293T supernatant and TZM-bl cells, 293T cell culture supernatants were added to 8 μm transwells (Greiner Bio-one), which were held in a 24-plate containing TZM-bl indicator cells. After 24-hrs of incubation of TZM-bl indicators cells with 293T cell culture supernatants, the TZM-bl indicator cells were trypsinized and plated as (15,000 cells/well/0.1ml) in 96-well plate in complete DMEM medium, and incubated for 24 hrs. The next day 10-fold dilutions of HIV-BaL virus and VSV-G pseudotyped scSIV virus were made and 10ul of the serially diluted viruses were added to the plated TZM-bl cells in a total volume of 50ul complete DMEM medium. Cells were then incubated for 2 hrs at 37°C. After incubation, an additional 50ul of complete DMEM medium was added to each well and the cells were incubated for 4 days at 37°C. For interferon blocking experiments, a total of 60 μg of anti-IFN-α or anti-IFN-β antibody (clones MMHA-2 and MAB814 respectively, from R&D Systems Inc.) was added to each well following infection. After 4 days, cells were washed once with PBS and the presence of β-galactosidase in the cell was determined using Pierce β-galactosidase assay kit (Thermo Fisher Scientific Inc.) and following the manufacturer’s instructions. Color development was terminated using the kit stop solution and the absorbance at 405 nm determined using a microplate spectrophotometer.

### Primary human CD4+ T cell virus inhibition assay

Venous blood in sodium heparin (Continental Blood Services, Inc., Miami, FL) was obtained as anonymous Buffy Coat donations from HIV-seronegative donors according to institutional review board approved protocols. Buffy coats were incubated with 1/10 volume of RosetteSep CD4+ T cell enrichment cocktail at room temperature for 20 min, diluted with one volume of PBS containing 2% human AB serum (Lonza Inc.). CD4+ T cells were separated from the remainder of PBMC and red blood cells by density gradient centrifugation. Purified CD4+ T cells were then washed twice with 2% Human AB serum in PBS and resuspended in RPMI 1640 medium supplemented with 2 mM glutamine, 100 U/ml penicillin, 100 μg/ml streptomycin, and 5% human AB serum (Lonza). Cells were adjusted to 1x10^6^/ml and activated with 5μg/ml phytohemagglutinin-P (PHA-P) (Sigma, St Louis, MO), seeded into 24-well plates and cultured at 37°C in 5% CO2 for 3 days. Activated cells were then washed with 1X PBS to remove PHA-P and cultured in RPMI supplemented medium. To evaluate virus inhibition, the activated CD4+ T cells were incubated with 293T cell culture supernatant in a transwell overnight on 24 well plates. Supernatant was generated from 293T cells previously transfected with pcDNA3.1 or ΔLMP1-MAVS plasmid at 37°C for 24 hours as described above. CD4+ T cells were then infected by washing the cells in RPMI once and adding 100 μl/well of RPMI supplemented media containing HIV-Bal virus at an MOI of 0.1 or 1, followed by culture at 37°C for 4 h. The cells were then washed twice with RPMI medium and fed with 2 ml of complete RPMI 1640 medium supplemented with 2 mM glutamine, 100 U/ml penicillin, 100 μg/ml streptomycin, 5% human AB serum and 20 units/ml of interleukin-2 (R&D systems). Cells were incubated at 37°C for 6 days. The culture supernatants from infected CD4+ T cells were collected at day 6 and stored at -80°C. The concentration of virus in the cell culture supernatant was determined by p24 antigen capture ELISA (Perkin Elmer, MA, USA).

### Real-time RT-PCR

Total RNA was isolated from 293T cell cultures transfected with pcDNA3.1, LMP1 or ΔLMP-MAVS with RNeasy Mini kit (Qiagen) and 500 ng of RNA were reverse transcribed with oligo(dT) primers using the Phusion RT-PCR kit (Thermo Scientific). Realtime PCR was performed with SYBR Green PCR Core Reagents (Applied Biosystems) and the following primer sets: Viperin (forward) 5-CGT GGA AGA GGA CAT GAC GGA AC-3, (reverse) 5-CCC TTT CTA CAG TTC AGA AAG CGC-3; ISG56 (forward) 5-CTG TCT TAC TGC ATC ACC AGA TAG G-3, (reverse) 5-CCC TCT AGG CTG CCC TTT TG-3; IFN-α (forward) 5-ACT TTG GAT TTC CCC AGG A-3, (reverse) 5-CAG GCA CAA GGG CTG TAT T-3; IFN-β (forward) 5-GAG CTA CAA CTT GCT TGG ATT CC-3, (reverse) 5- CAA GCC TCC CAT TCA ATT GC-3; IP-10 (forward) 5-TGA AAT TAT TCC TGC AAG CCA ATT-3, (reverse) 5-CAG ACA TCT CTT CTC ACC CTT CTT T-3; MIP-1β (forward) 5-GTC TGT GCT GAT CCC AGT GA-3, (reverse) 5-GGA CAC TTA TCC TTT GGC TA-3; RANTES (forward) 5- CCG CGG CAG CCC TCG CTG TCA TCC-3, (reverse) 5-CAT CTC CAA AGA GTT GAT GTA CTC C-3; housekeeping genes: β-actin (forward) 5-AGC CTC GCC TTT GCC GA-3, (reverse) 5- CTG GTG CCT GGG GC G-3; and GAPDH (forward) 5-GGC TGA GAA CGG GAA GCT T-3, (reverse) 5-AGG GAT CTC GCT CCT GGA A-3. Each sample was assayed in triplicate with the ABI StepOne Detection System (Applied Biosystems) for 45 cycles of 15 s at 95°C followed by 1 min at 60°C and a final dissociation protocol to screen for false amplification products. The value obtained for the pcDNA3.1 transfected control sample was set to 1 and changes in mRNA expression with LMP1 or ΔLMP1-MAVS are given in relation to the pcDNA3.1 control.

### Gene Microarray Analysis

293T cells were transfected with pcDNA3.1 or ΔLMP1-MAVS DNA for 36 hrs in triplicate and total cellular RNA was extracted using the RNeasy Mini kit (Qiagen). Human CD4+ T cells were isolated by negative selection from 3 healthy donors as described above and the 3 independent samples were incubated with 293T cell culture supernatant collected after transfection with pcDNA3.1 or ΔLMP1-MAVS. CD4+ T cells were cultured with 293T supernatant in a transwell as described above for 24 hrs at 37°C and total cellular RNA was extracted using the RNeasy Mini kit (Qiagen). Gene microarray expression data was obtained by hybridization of total RNA to the Illumina HumanHT-12_V4_0_R1_15002873_B platform. Data was loaded on GeneSpring^™^ 13.0 (Agilent Tech. CA, USA) and was subjected to rigorous quality control. All 47230 probes were subjected to an unpaired Student’s t-test between the pcDNA and ΔLMP1-MAVS in both 293T and CD4+ T cell conditions. Multiple testing corrections were performed on p-values using Benjamini-Hochberg method and genes were considered to be significant for p-values ≤ 0.05 and Fold change ≥ 2. Hierarchical clustering was performed on significantly differentially expressed genes in both cell conditions using ‘Euclidean’ distance metric and ‘Complete’ linkage rule. All microarray data files are available through the NCBI GEO repository as record GSE65541.

### Isolation of exosomes by ultracentrifugation and infection assays

293T cells were transfected with pcDNA3.1, LMP1 or ΔLMP1-MAVS plasmid DNA as described above in methods. Cell culture supernatant was collected 48hr after transfection and tissue culture supernatants (25 ml) were centrifuged for 30 minutes at 2,000×g to pellet cells. The supernatant was transferred to a fresh tube and centrifuged at 10,000×g for 30 minutes at 4°C. Filtering was performed after clearance of cellular debris and prior to ultracentrifugation where noted. Supernatants were transferred to ultracentrifuge tubes and spun in a Sorvall AH-629 swinging bucket rotor for 70 minutes at 100,000×g. The supernatant was stored and described as exosomes-depleted supernatant. The pellet was resuspended in 25 ml of sterile PBS and passed through a 0.2-micron filter. Exosomes were centrifuged at 110,000×g for an additional 70 minutes to wash. The supernatant was discarded and the resultant pellet was resuspended in 0.2 ml of sterile PBS and used for subsequent experiments.

For HIV inhibition assays using exosomes or exosomes-depleted supernatants, 100ul of exosomes or exosomes-depleted supernatant were added to the TZM-bl indicator cell in 24-well plate in 500ul of complete DMEM medium and incubated for 24 hrs at 37°C. After 24-hrs of incubation, the TZM-bl indicator cells were trypsinized and plated as (15,000 cells/well/0.1ml) in 96-well plate in DMEM and 10% FBS medium and incubated for 24 hrs. Next day, 10-fold dilutions of HIV-Bal virus were made and 10ul of the serially diluted viruses were added to the plated TZM-bl cells in a total volume of 50ul. Cells were incubated for 2 hrs at 37°C. After incubation, 50ul of culture medium was added to each well and the cells were incubated for 4 days at 37°C. The cells were washed once with PBS and the presence of β-galactosidase in the cell was determined using a Pierce β-galactosidase assay kit (Thermo Fisher Scientific Inc.) following the manufacturer’s instructions. Color development was terminated using the kit stop solution and absorbance at 405 nm determined using a microplate spectrophotometer.

### ELISA assay

Supernatant from transfected 293T cells and transduced MDDC was analyzed by ELISA assay for IFN-α and IFN-β using Verikine human IFN-α and IFN-β ELISA kits (PBL Inc.). Samples were assayed in triplicate at various dilutions and interferon concentrations were determined based on a standard curve following manufacturers instructions.

### Production of recombinant adenovirus

Replication deficient adenovirus constructs (AdEasy or Adeno-X Tet-On 3G), were made as described by the manufacturers (Agilent and Clontech respectively). Replication deficient adenovirus (pAdEasy-1), containing GFP or codon-optimized HIV-1 Gag IIIB, were constructed as described by the manufacturer protocol (AdEasy Adenoviral vector system, Agilent tech). Genes were PCR amplified and cloned into the pAdenoVator-CMV5 shuttle vector (Qbiogene). Clones were sequenced to select a clone for virus generation. The CMV5-shuttle vectors were electroporated into BJ5183 (Agilent tech) cells containing the pAdEasy-1 plasmid to generate recombinants. The recombined AdEasy viral vectors were then linearized and transfected into AD293 cells (Stratagene). Recombinant Ad5 virus was purified from AD293 cells and concentrated using the Adeno-X Mega purification kit (Clontech).

Adenoviruses expressing LMP1 or ΔLMP1-MAVS were constructed using the Adeno-X Tet-On 3G inducible system (Clontech). Both LMP1 and ΔLMP1-MAVS constructs were cloned to include an IRES-GFP sequence, allowing LMP1 or ΔLMP1-MAVS protein expression to be tracked by GFP flurescence. LMP1-IRES-GFP and ΔLMP1-MAVS-IRES-GFP were cloned into the Adeno-X system as described by the manufacturer. Following PCR of the complete gene, LMP1-IRES-GFP or ΔLMP1-MAVS-IRES-GFP was cloned into a linearized Ad5-Tet-On genome using the InPhusion cloning kit (Clontech). Ligated plasmid was grown in Stellar competent cells (Clontech). Following sequencing to confirm the correct gene sequence, viral vectors were linearized and transfected into AD293 cells (Stratagene). Following propagation in AD293 cells, recombinant Ad5 virus was purified and concentrated using the Adeno-X Mega purification kit (Clontech). To determine infectious colony forming units (CFU), viruses were titered using the Adeno-X Rapid titer kit (Clontech).

### DC activation and maturation assay

Monocyte derived DC were generated by standard methods [38] from PBMC obtained from anonymous donors (Continental Blood Services Inc, Miami) and plated on 6 well plates. DC were then transduced with Ad5-ΔLMP1-MAVS or Ad5-Gag control (MOI = 50). DC were incubated with virus at 4°C for 1 h, followed by 3 h at 37°C. Complete media was then added to 2ml. As a positive control, DC were matured with cytokine mix Mimic (5 ng/ml TNF-α (Peprotech), 5ng/ml IL-1b (Peprotech), 750ng/ml IL-6 (Peprotech), and 1ug/ml PGE2 (Sigma)). Cells were incubated for 36 hours at 37°C, harvested, and stained with the following antibodies: CD86 clone 2331 (FUN-1), CD80 clone L307.4, HLA-DR clone TU36, CD83 clone HB15e, CD40 clone 5C3, CD197 (CCR7) clone 3D12, and CD11c clone 3.9) (BD Bioscience). After flow cytometry analysis, the mean fluorescence intensity (MFI) for each antibody was calculated for CD11c+ dendritic cells under each experimental condition. FlowJo 7.6.4 flow cytometry analysis software (FlowJo, Ashland, OR) was used for analysis. Three independent wells were analyzed for each condition.

#### Cytokine analysis

For cytokine measurement, 36-hour supernatants from DC cultures described above were analyzed by cytometric bead array using the human inflammation kit (BD Biosciences, San Jose, CA), following manufacturers instructions. Cytokines analyzed included IL-8, IL-1β, IL-10, IL-6, TNF-α, and IL-12p70.

#### Vaccination and vaccinia virus challenge

Female BALB/c mice (7–8 weeks old) (Harlan Inc.) were used in all vaccination experiments. Animals were housed at the University of Miami under the guidelines of the National Institutes of Health (NIH, Bethesda, MD). All animal experiments were performed in accordance with national and institutional guidance for animal care and were approved by the IACUC of the University of Miami. Mice were immunized (five mice per group) with Ad5 virus by intramuscular injection in both hind quadriceps muscles. Mice (5 per group) were injected with 1X10^6^ infectious units (IFU) of AD-5-Gag and 1X10^6^ IFU of Ad5-GFP, Ad5-LMP1 or Ad5-ΔLMP1-MAVS. 1X10^6^ IFU corresponded to 1X10^9^ vp for vaccine optimization experiments. Ad5-GFP was used as a negative control. One month after immunization, mice were challenged i.p with 1x10^7 vp vaccinia-Gag virus vP1287 (NIH AIDS Research and Reference Reagent Program). Five days after challenge, mice were sacrificed and both ovaries and fallopian tubes were removed and homogenized in 500ul PBS. For measurement of virus titers, samples were sonicated, and evaluated in triplicate by 10-fold serial dilution on CV-1 cells (American Type Culture Collection) plated in 24 well plates. After 48-hour incubation, plates were stained with 0.1% (w/v) crystal violet in 20% ethanol. Plaques were counted to determine PFU of virus.

### Statistical analysis

All statistical analysis was performed using GraphPad Prism 6 software. TZM-bl viral inhibition data was analyzed by two-way ANOVA using Tukey’s multiple comparisons test. Please refer to gene microarray section above for microarray statistical analysis methods. For all other data, a one-way ANOVA was performed to determine significance, followed by a two-tailed Student's *t*-test. All p values are labeled by asterisks denoting p < 0.05 (*), p < 0.01 (**), or p < 0.001 (***).

## Results

### Construction, expression and in vitro activity of ΔLMP1-MAVS fusion protein

**Fig 1:**
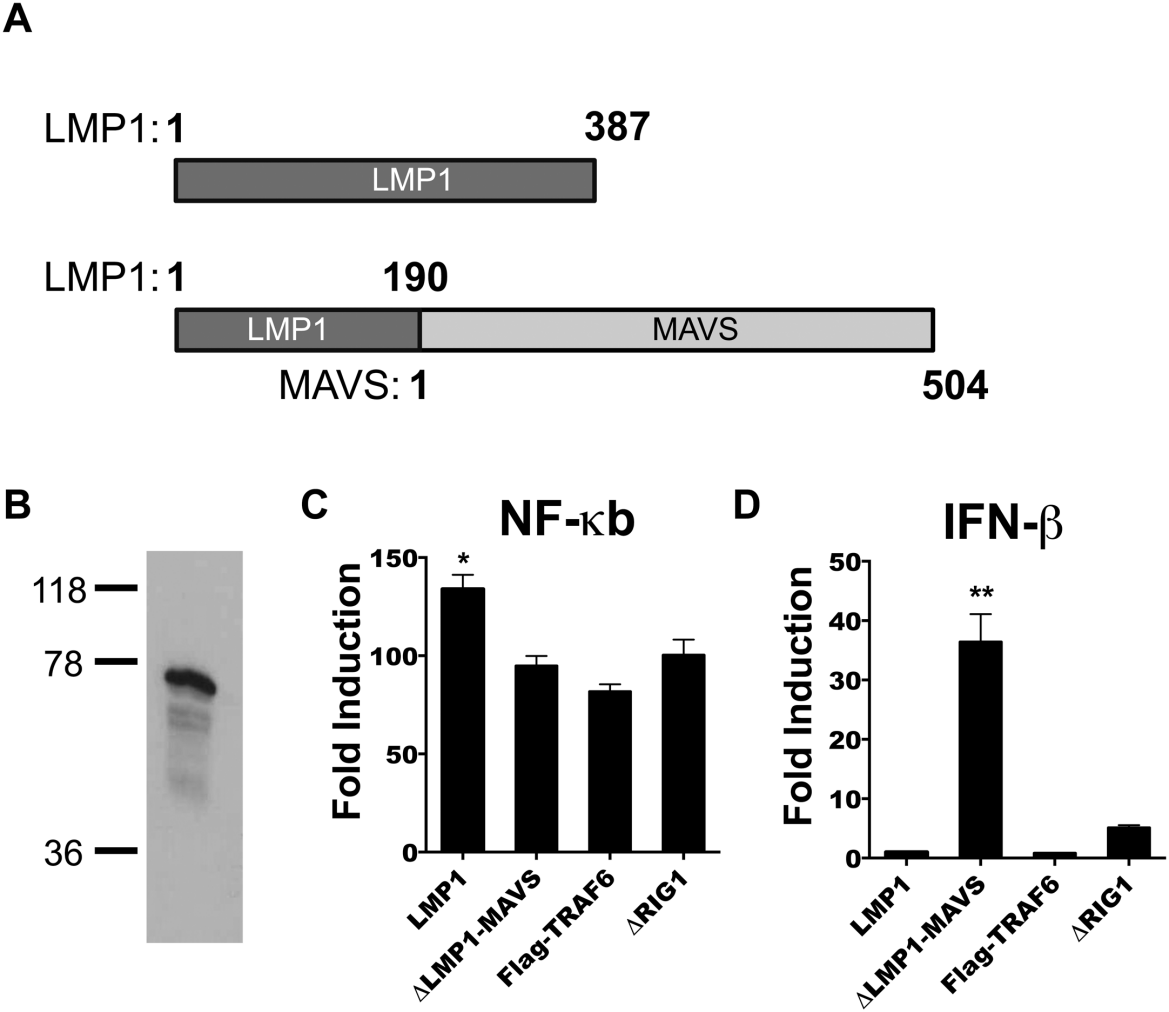
Construction and in vitro activity of ΔLMP1-MAVS. (A) Cloning strategy for vectors expressing full length LMP1 or ΔLMP1-MAVS fusion. (B) Western blot analysis of lysate from 293T cells transfected with ΔLMP1-MAVS plasmid. Luciferase activity assay was performed on 293T cells co-transfected with NF-κB (C) or IFN-β (D) luciferase reporter constructs and constructs expressing LMP1, ΔLMP1-MAVS, Flag-TRAF6, or ΔRIG-I. Fold induction was measured relative to empty vector pcDNA3.1. *p<0.05, **p<0.01.

To generate a constitutively active MAVS recombinant protein, full length MAVS (amino acids 1–504) was fused to the first 190 amino acids of LMP1, generating the recombinant gene ΔLMP1-MAVS (Fig 1A). This construct was cloned into the expression vector pcDNA3.1. Western blot of transfected 293T cells was performed to confirm expression of ΔLMP1-MAVS. As shown in Fig 1B, a band at 76 kDa was observed using an anti-MAVS antibody. This corresponded to the theoretical weight of ΔLMP1-MAVS (75.3 kDa). To assess the ability of ΔLMP1-MAVS to induce NF-κB and IFN-β promoter mediated gene expression, ΔLMP1-MAVS or full length LMP1 were co-transfected along with NF-κB or IFN-β firefly luciferase reporter plasmids into 293T cells. Induction by LMP1 or ΔLMP1-MAVS was compared to either a positive control NF-κB inducing plasmid (Flag-TRAF6) or a positive control IFN-β inducing plasmid (ΔRIG-I). As shown in Fig 1C, LMP1 generated significantly higher levels of NF-κB-mediated luciferase induction compared to ΔLMP1-MAVS or the Flag-TRAF6 control (p<0.05). In contrast, ΔLMP1-MAVS generated significantly higher IFN-β induction compared to all constructs (Fig 1D). ΔLMP1-MAVS gave a mean 36-fold induction of IFN-β, compared to ΔRIG-I that gave a mean 5-fold induction, suggesting that ΔLMP1-MAVS is a superior inducer of high levels of type I interferon.

### ΔLMP1-MAVS inhibits HIV-1 and SIV replication

**Fig 2:**
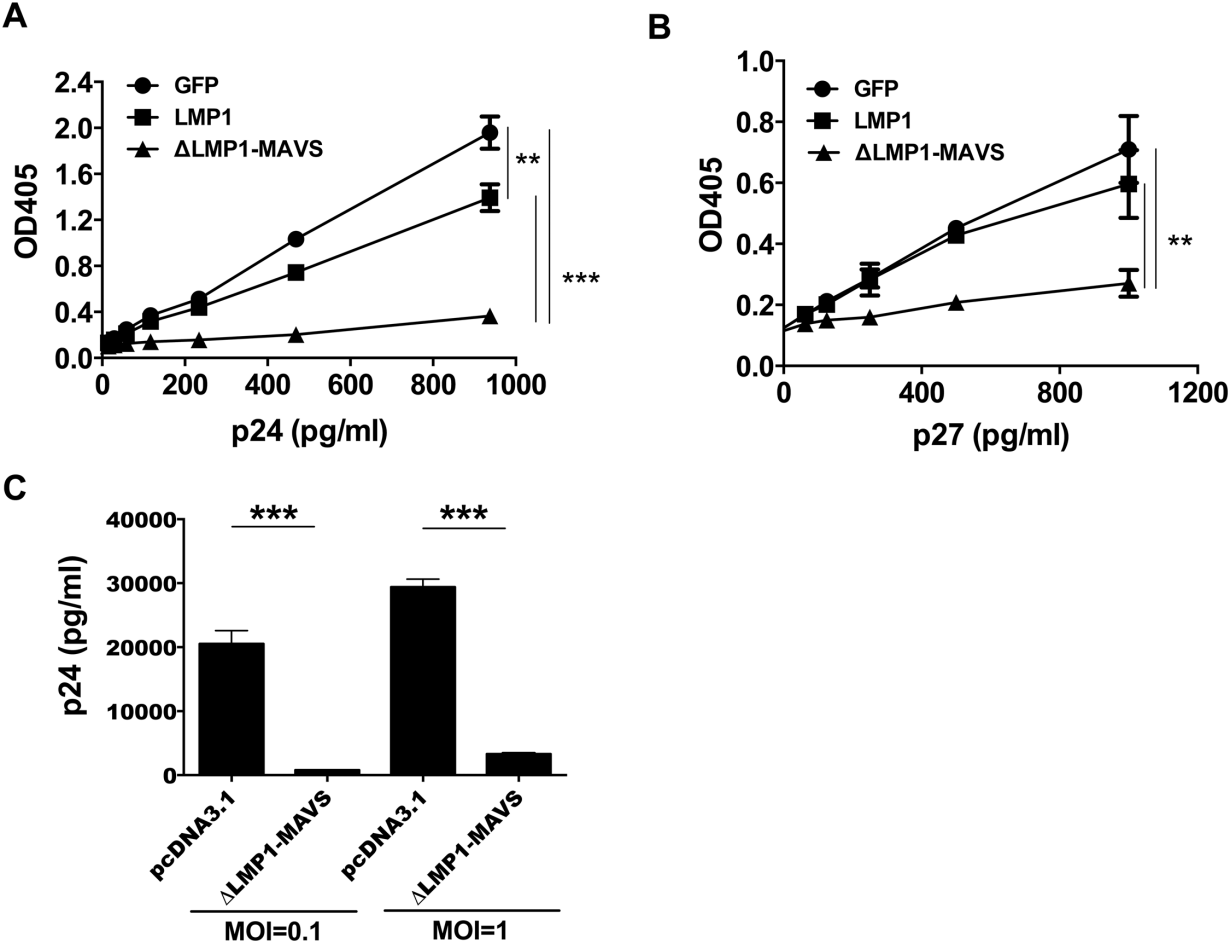
Inhibition of HIV and SIV viral infection of TZM-bl and primary human CD4+ T cells. The relative level of viral replication in TZM-bl cells was measured following incubation with supernatant from 293T cells transfected with pcDNA3.1 vector expressing GFP (control), LMP1, or ΔLMP1-MAVS plasmid. (A) Infection with HIV-1 BaL strain. (B) Infection with VSV-G pseudotyped single cycle SIV. (C) CD4+ T cells were isolated from a healthy donor by negative selection, activated, and cultured with 293T supernatant for 24 hours. Cells were then washed and infected with HIV-1 BaL at an MOI or 0.1 or 1. The concentration of p24 was measured 6 days later by ELISA assay. *p<0.05, **p<0.01, ***p<0.001.

**Fig 3:**
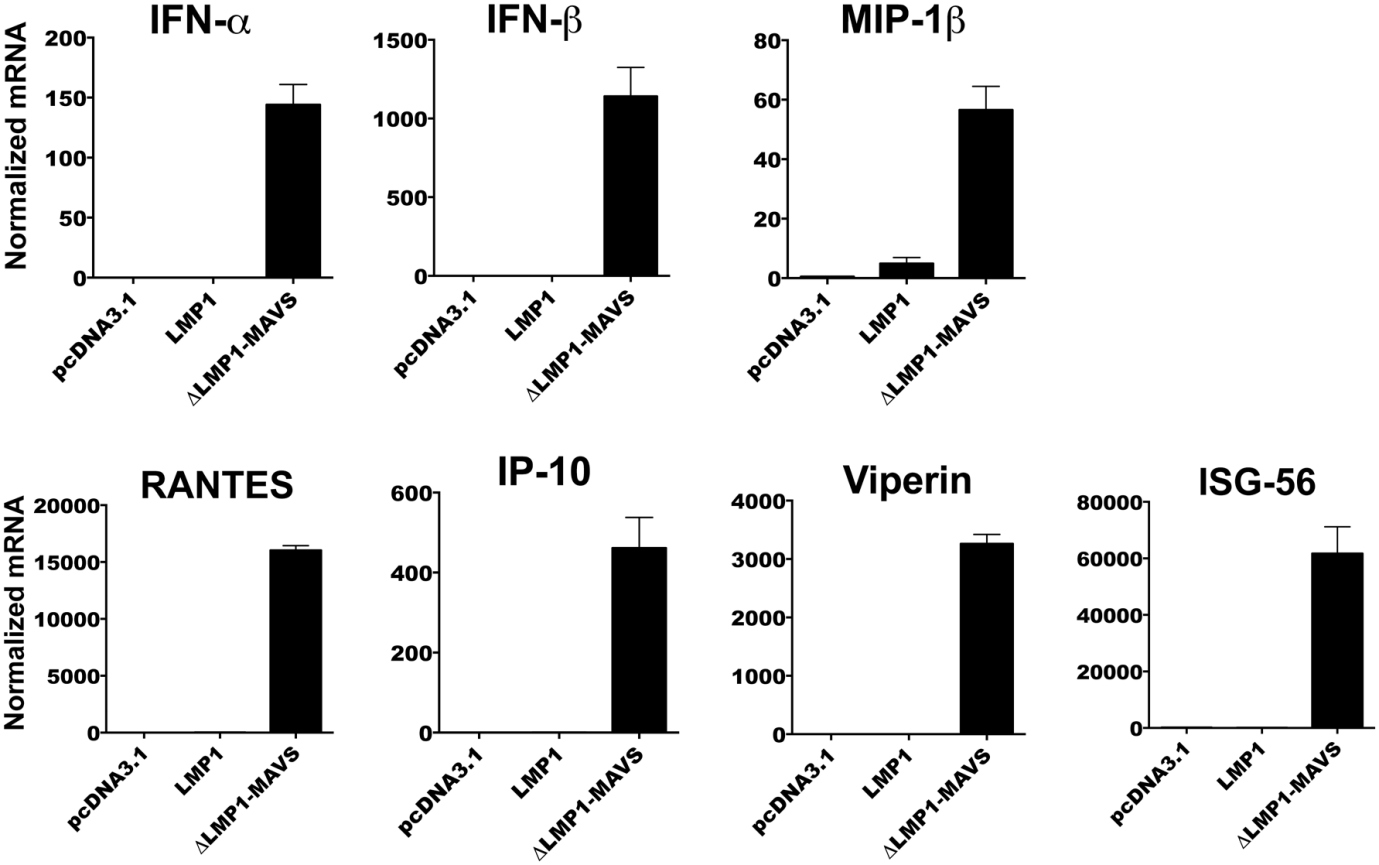
RealTime PCR analysis of 293T cells transfected with LMP1 or ΔLMP1-MAVS. 293T cells were transfected with pcDNA3.1, LMP1 plasmid, or ΔLMP1-MAVS plasmid and total RNA isolated following 36-hour culture. Normalized expression was determined relative to the pcDNA3.1 control.

Previous studies have shown that type I interferon impacts HIV-1 viral replication in vitro [42,43]. Given the high level of type-I interferon expression following ΔLMP1-MAVS transfection, we therefore asked whether soluble factors generated by ΔLMP1-MAVS-transfected 293T cells could directly impact HIV-1 replication. TZM-bl HIV-1 replication reporter cells were cultured with supernatant from 293T cells transfected with pcDNA3.1-GFP (control), LMP1 or ΔLMP1-MAVS plasmid DNA. 293T supernatant was either placed in a transwell (Fig 2) or added directly to the TZM-bl cells (data not shown), and gave equivalent results. Following 24-hour incubation with 293T supernatant, TZM-bl cells were washed and infected with HIV-1 BaL at a range of virus concentrations. As shown in Fig 2A, ΔLMP1-MAVS-transfected 293T supernatant significantly reduced viral replication when compared to GFP or LMP1. LMP1 modestly inhibited viral replication compared to GFP, but only at higher viral titers. To confirm these results using an alternative lentiviral system, TZM-bl cells were infected with single cycle SIV pseudotyped with VSV-G. Again, we observed a reduction in viral replication with ΔLMP1-MAVS, but not LMP1 (Fig 2B). These data confirmed that viral inhibition is independent of HIV-1 envelope-mediated cell entry, and that a soluble factor generated by ΔLMP1-MAVS-transfected 293T cells can inhibit both HIV-1 and SIV replication. While these data were encouraging, it is known that cell lines such as HeLa-derived TZM-bl cells may be more sensitive to viral inhibition when compared to primary CD4+ T cells [43–45]. Therefore, human CD4+ T cells were isolated by negative selection from an uninfected donor, activated with PHA, cultured overnight with fresh supernatant from 293T cells transfected with either pcDNA3.1 control plasmid or ΔLMP1-MAVS plasmid, washed, and then infected with HIV-1 BaL. As shown in Fig 3C, supernatant from 293T cells expressing ΔLMP1-MAVS significantly inhibited CD4+ T cell virus production as measured by p24 ELISA. Inhibition was observed following infection with either low titer (MOI = 0.1) or high titer (MOI = 1) virus.

### ΔLMP1-MAVS markedly upregulated expression of type I interferons, CCR5 chemokines, and antiviral ISG

To determine potential mechanisms of HIV-1 restriction, realtime PCR was performed on mRNA from 293T cells transfected with empty vector (pcDNA3.1), pLMP1, or pΔLMP1-MAVS. As shown in Fig 3, ΔLMP1-MAVS upregulated the expression of IFN-α, IFN-β, MIP-1β, RANTES, IP-10 and the ISGs Viperin and ISG56. In contrast, LMP1 did not upregulated these genes, highlighting the unique gene expression profile of the ΔLMP1-MAVS fusion. While LMP1 did not upregulate type I interferon or ISG, previous studies in our laboratory have shown that LMP1 can highly upregulate downstream markers of NF-κB stimulation, including IL-6 [38].

### Microarray analysis of cells stimulated with ΔLMP1-MAVS in cis and in trans

**Fig 4:**
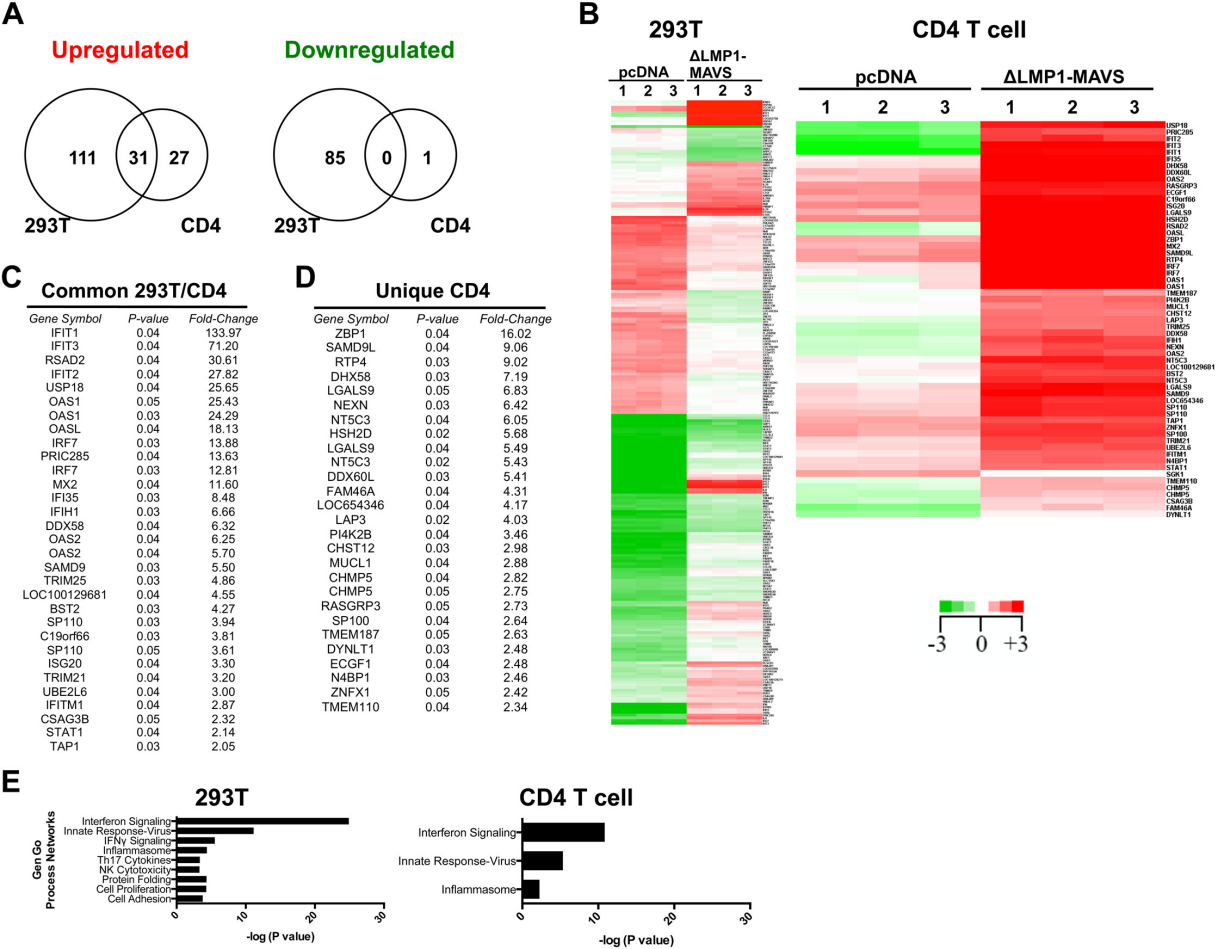
Gene array analysis of transfected 293T cells and primary CD4+ T cells cultured with 293T supernatant. Three independent wells of 293T cells were transfected with pcDNA3.1 empty vector or ΔLMP1-MAVS plasmid and total RNA isolated 36 hours later. Primary CD4+ T cells from 3 independent donors were cultured with 293T supernatant (collected 24 hours following pcDNA3.1 or ΔLMP1-MAVS transfection). Total CD4+ T cell RNA was isolated 36 hours later. (A) Venn Diagrams of the number of probe sets upregulated and down-regulated (>2-fold change) by ΔLMP1-MAVS. (B) Differential gene expression of 293T cells and CD4+ T cells. (C) List of genes upregulated by ΔLMP1-MAVS in both 293T cells and CD4+ T cells. Fold-change and P-values for each probe set are shown for CD4+ T cells following ΔLMP1-MAVS treatment. (D) List of genes upregulated by CD4+ T cells but not 293T cells. Fold-change between pcDNA3.1 and pΔLMP1-MAVS and P-values for each probe set are shown. (E) Gen Go networks analysis of pathways significantly upregulated by ΔLMP1-MAVS in transfected 293T cells, or CD4+ T cells cultured with ΔLMP1-MAVS transfected 293T supernatant.

Next, overall gene expression was evaluated by microarray analysis of 293T cells transfected with ΔLMP1-MAVS or empty vector. We also evaluated primary human CD4+ T cells from 3 independent donors cultured with transfected 293T cells in a trans well assay. We directly compared 293T cells transfected with ΔLMP1-MAVS to 293T cells transfected with pcDNA3.1 empty vector. Similarly, we compared CD4+ T cells cultured with ΔLMP1-MAVS-transfected 293T cell supernatant to CD4+ T cells cultured with supernatant from empty vector-transfected 293T cells. As shown in Fig 4A, 293T cells differentially regulated a total of 227 targets in the presence of ΔLMP1-MAVS. Upregulated pathways included those associated with innate immune responses to RNA viral infection, inflammation, and type I interferon signaling. Similarly, CD4+ T cells co-cultured with ΔLMP1-MAVS-transfected 293T supernatant differentially regulated 59 targets including 56 genes (Fig 4B). 28 genes were significantly upregulated in both CD4+ T cells and 293T cells (Fig 4C), 27 genes were significantly upregulated in CD4+ T cells but not 293T cells, and 1 gene (SGK1) was significantly downregulated in CD4+ T cells, but not 293T cells. Analysis of overlapping genes revealed upregulation of IRF7 in both 293T cells and CD4+ T cells, as well as the upregulation of a number of ISG including Viperin/RSAD2 (31-fold), ISG56/IFIT1 (28- to 130-fold), Tetherin/BST2 (4-fold), ISG54/IFIT2 (28-fold), IFITM1 (3-fold), ISG20 (3-fold), and MxB/MX2 (12-fold). Both CD4+ T cells and 293T cells also upregulated a number of genes associated with innate immune responses to RNA viral infection, including IRF7 (14-fold), RIG-I (6-fold) and MDA5 (7-fold). Similarly, we observed upregulation of a number of genes involved in type I interferon signaling, including STAT1. As shown in Fig 4D, a number of genes were significantly upregulated by CD4+ T cells but not 293T cells, including RTP4 (9-fold), ZBP1 (16-fold) and SAMD9L (9-fold).

### Inhibition assays with purified exosomes or exosome depleted supernatant

**Fig 5:**
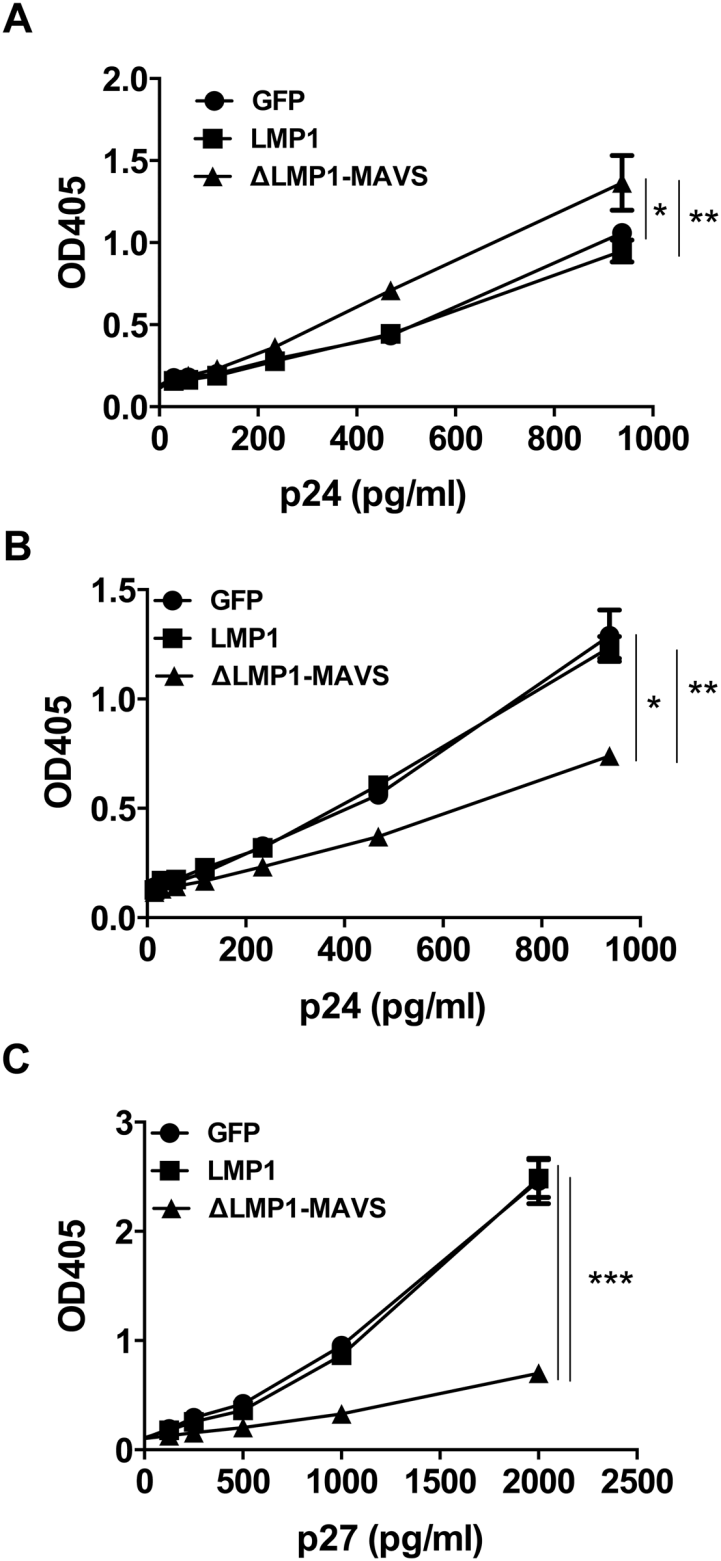
Exosome-depleted 293T supernatant inhibits HIV and SIV replication. 293T cells were transfected with pcDNA3.1 plasmids encoding GFP, LMP1, or ΔLMP1-MAVS and supernatant was isolated. Supernatant was depleted of exosomes by ultracentrifugation. (A) TZM-bl cells were cultured with isolated exosomes and infected with increasing concentrations of HIV-1 BaL. (B) TZM-bl cells were cultured with exosome-depleted supernatant, followed by infection with HIV-1 BaL. (C) TZM-bl cells were cultured with exosome-depleted supernatant, followed by infection with VSV-G pseudotyped single cycle SIV. *p<0.05, **p<0.01, ***p<0.001.

LMP1 is known to induce exosomes [46], providing a potential mechanism for delivering HIV-1 inhibition factors to neighboring cells. To test this, exosomes were purified from 293T supernatants by ultracentrifugation and cultured with TZM-bl cells followed by HIV-1 BaL infection. As shown in Fig 5A, we observed comparable levels of HIV-1 replication for both GFP (control) and LMP1 transfected 293T supernatant. Surprisingly, ΔLMP1-MAVS derived exosomes showed a modest increase in viral replication, suggesting that exosomes from ΔLMP1-MAVS-transfected 293T cells may contain factors that enhance HIV-1 replication. In contrast, exosome-depleted supernatant from ΔLMP1-MAVS transfected 293T cells significantly inhibited replication of HIV-1 BaL (Fig 5B) and single cycle SIV (Fig 5C), suggesting that exosomes do not mediate inhibition of lentiviral replication.

### Type I interferon blockade

**Fig 6:**
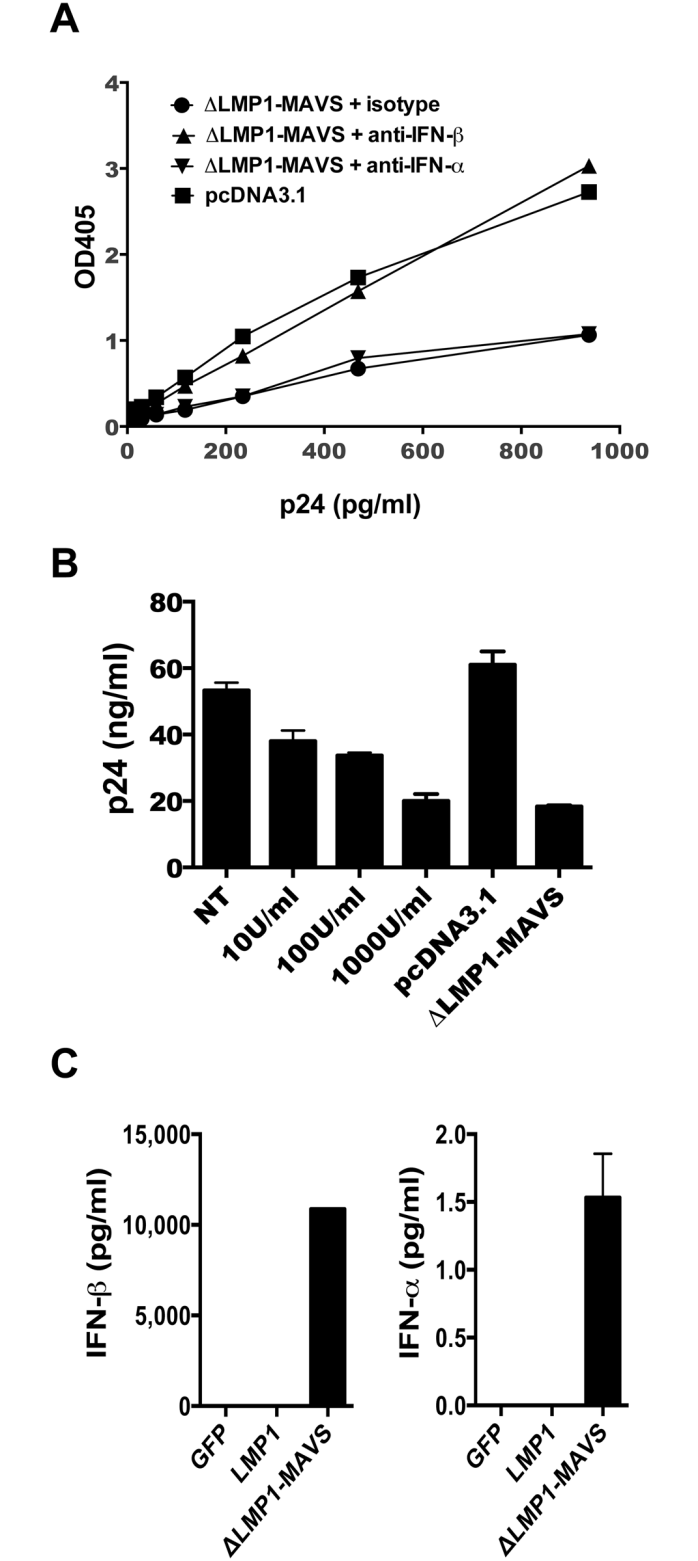
Beta-interferon mediates inhibition of HIV-1 BaL replication. (A) The relative level of HIV-1 BaL strain viral replication in TZM-bl cells was measured following incubation with supernatant from 293T cells transfected with either pcDNA3.1 or pΔLMP1-MAVS plasmid, combined with 60 μg of isotype control antibody, anti-IFN-β antibody, or anti-IFN-α antibody. (B) Human CD4+ T cells were infected with HIV-1 BaL in the presence of increasing concentrations of interferon-α and compared to infection in the presence of 293T supernatant following transfection with either pcDNA3.1 or pΔLMP1-MAVS plasmid. (C) Supernatant from 293T cells transfected with pcDNA3.1 GFP, LMP1, or ΔLMP1-MAVS plasmid was assayed for IFN-α and IFN-β secretion by ELISA. NT: no treatment.

Previous studies have shown that type I interferon directly inhibits HIV-1 replication [42,47]. Therefore we decided to investigate the role of type I interferons on the viral inhibition we observed. A TZM-bl transwell assay was performed in the presence of 60μg of anti-interferon antibodies. As shown in Fig 6A, anti-IFN-β antibody abrogated the antiviral activity induced by ΔLMP1-MAVS. Surprisingly, anti-IFN-α antibody was unable to reduce inhibition, suggesting that IFN-β alone was mediating this response. To confirm that HIV replication in CD4+ T cells could be inhibited by IFN-α, primary CD4+ T cells were infected with HIV-1 at an MOI of 0.1 in the presence of increasing doses of recombinant interferon alpha. As shown in Fig 6B, IFN-α inhibited HIV-1 virus production in a dose-dependent manner, with equivalent levels of inhibition at 1,000 U/ml IFN-α when compared to ΔLMP1-MAVS-transfected 293T cell supernatant. Finally, we measured IFN-α and IFN-β levels in the 293T supernatant by ELISA. As shown in Fig 6C, ΔLMP1-MAVS transfection of 293T cells induced high levels (>10ng/ml) of secreted IFN-β, but only minimal levels of secreted IFN-α (<2 pg/ml).

### Dendritic cells activation and maturation following ΔLMP1-MAVS transduction

**Fig 7:**
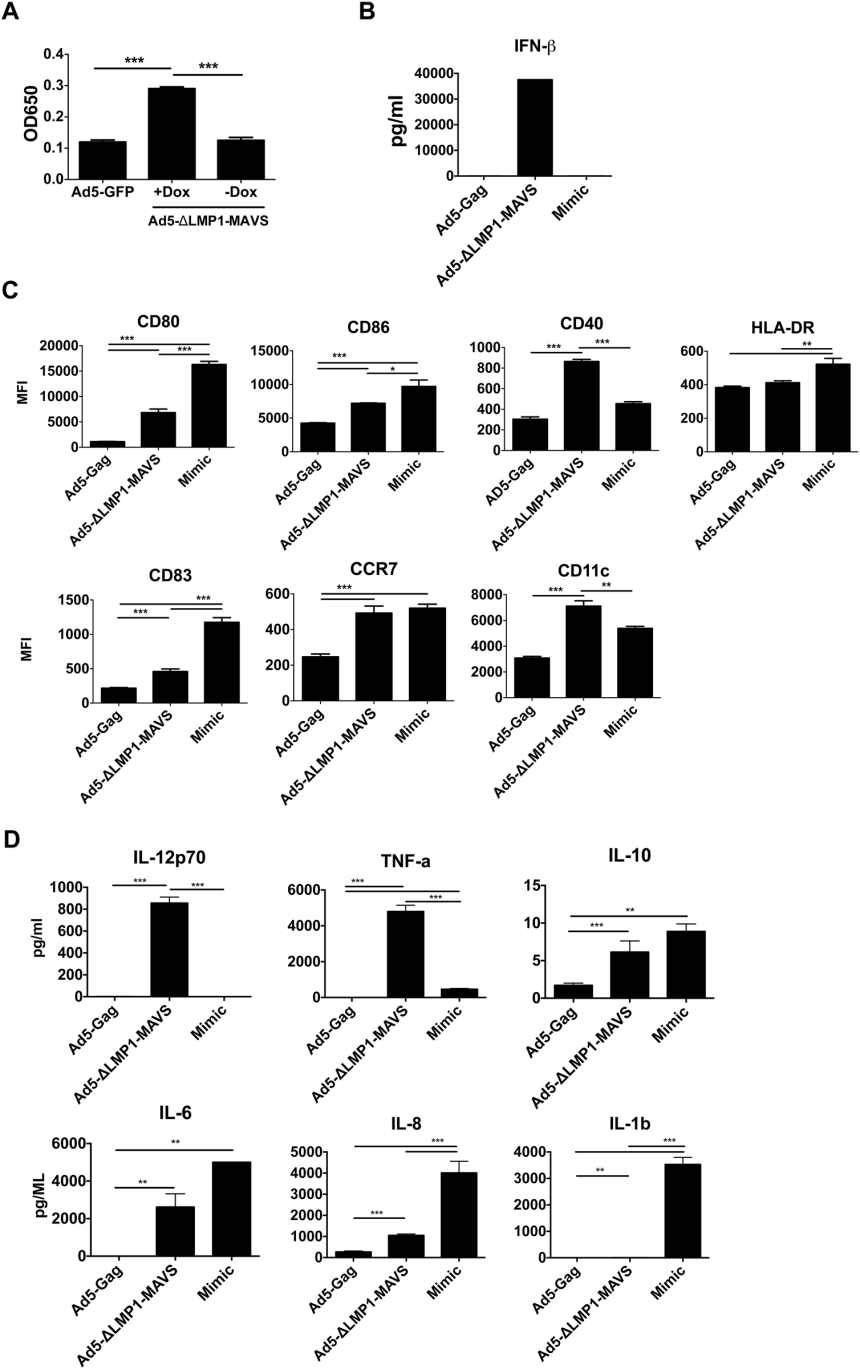
Activation and maturation of human DC following transduction with Ad5-ΔLMP1-MAVS. (A) The biological activity of an adenoviral vector (Ad5) expressing ΔLMP1-MAVS was measured by transduction into HEK-293 cells expressing a secreted alkaline phosphatase (SEAP) reporter under control of NF-κB. Relative SEAP activity was measured at 650nm. Activity was measured in the presence or absence of doxycycline and compared to transduction with control Ad5-GFP virus. (B) IFN-β ELISA assay was performed on supernatant following 36-hour infection of human monocyte derived dendritic cells (DC) with Ad5-ΔLMP1-MAVS, negative control virus (Ad5-Gag), or incubation of DC with positive control cytokine mix (Mimic). (C) DC phenotypic markers were measured by flow cytometry following transduction of DC with Ad5-ΔLMP1-MAVS, Ad5-Gag, or Mimic. DC were analyzed after 36-hour culture. (D) Cytometric bead array analysis was performed on supernatant collected from DC after 36-hour culture. *p<0.05, **p<0.01, ***p<0.001.

MAVS signaling induced by RIG-I or MDA-5 agonists is known to play a critical role in the activation of dendritic cells [48,49]. To evaluate DC activation in the context of ΔLMP1-MAVS, we constructed an Ad5 adenoviral vector expressing ΔLMP1-MAVS under the control of a Tet-On promoter. To confirm biological activity of Ad5-ΔLMP1-MAVS, we evaluated NF-κB mediated signaling in the presence or absence of doxycycline. An HEK-293 cell line expressing secreted alkaline phosphatase under the control of NF-κB [50] was transfected with Ad5-GFP control or Ad5-ΔLMP1-MAVS in the presence or absence of doxycycline. As shown in Fig 7A, in the presence of doxycycline, Ad5-ΔLMP1-MAVS transduced cells secreted significantly higher levels of SEAP compared to Ad5-ΔLMP1-MAVS transduced cells in the absence of doxycycline or control cells. Next, DC were transduced with Ad5-ΔLMP1-MAVS or a control Ad5-Gag vector. As shown in Fig 7B, ΔLMP1-MAVS induced high levels of IFN-β secretion from human monocyte derived DC, as measured by ELISA assay, when compared to Ad5-GFP transduced DC or DC matured with a Mimic positive control. As shown in Fig 7C, ΔLMP1-MAVS significantly increased a number of DC activation markers, including CD80, CD86, and CD40. Interestingly, we did not observe increased levels of HLA-DR following ΔLMP1-MAVS transduction. Mimic further increased CD80, CD86, CD83 and HLA-DR MFI compared to ΔLMP1-MAVS, suggesting that ΔLMP1-MAVS does not optimally induce these markers. In contrast, ΔLMP1-MAVS significantly increased the maturation markers CD40 and CD11c compared to Mimic. Ad5-ΔLMP1-MAVS also significantly increased the DC maturation marker CCR7 to levels similar to Mimic. Cytokine secretion from dendritic cell cultures was also evaluated. Ad5-ΔLMP1-MAVS transduced DC secreted high levels of IL-12p70 (>800 pg/ml) compared to both Ad5-Gag and Mimic (<10 pg/ml) (Fig 7D). In a separate study, transduction of DC with Ad5-LMP1 induced secretion of low levels of IL-12p70 (<10 pg/ml, data not shown), highlighting the potent induction of IL-12p70 we observed with ΔLMP1-MAVS. Similarly, ΔLMP1-MAVS induced secretion of high levels of TNF-α (>4,000 pg/ml). Compared to Ad5-Gag, Ad5-ΔLMP1-MAVS induced low but significant levels of IL-10 (~5 pg/ml), at levels similar to the Mimic control. Ad5-ΔLMP1-MAVS also induced significant secretion of IL-1β, IL-6 and IL-8 compared to Ad5-Gag, but at lower levels when compared to Mimic.

### Δ**LMP1-MAVS is an effective molecular adjuvant in a mouse HIV-1 vaccine model**

**Fig 8:**
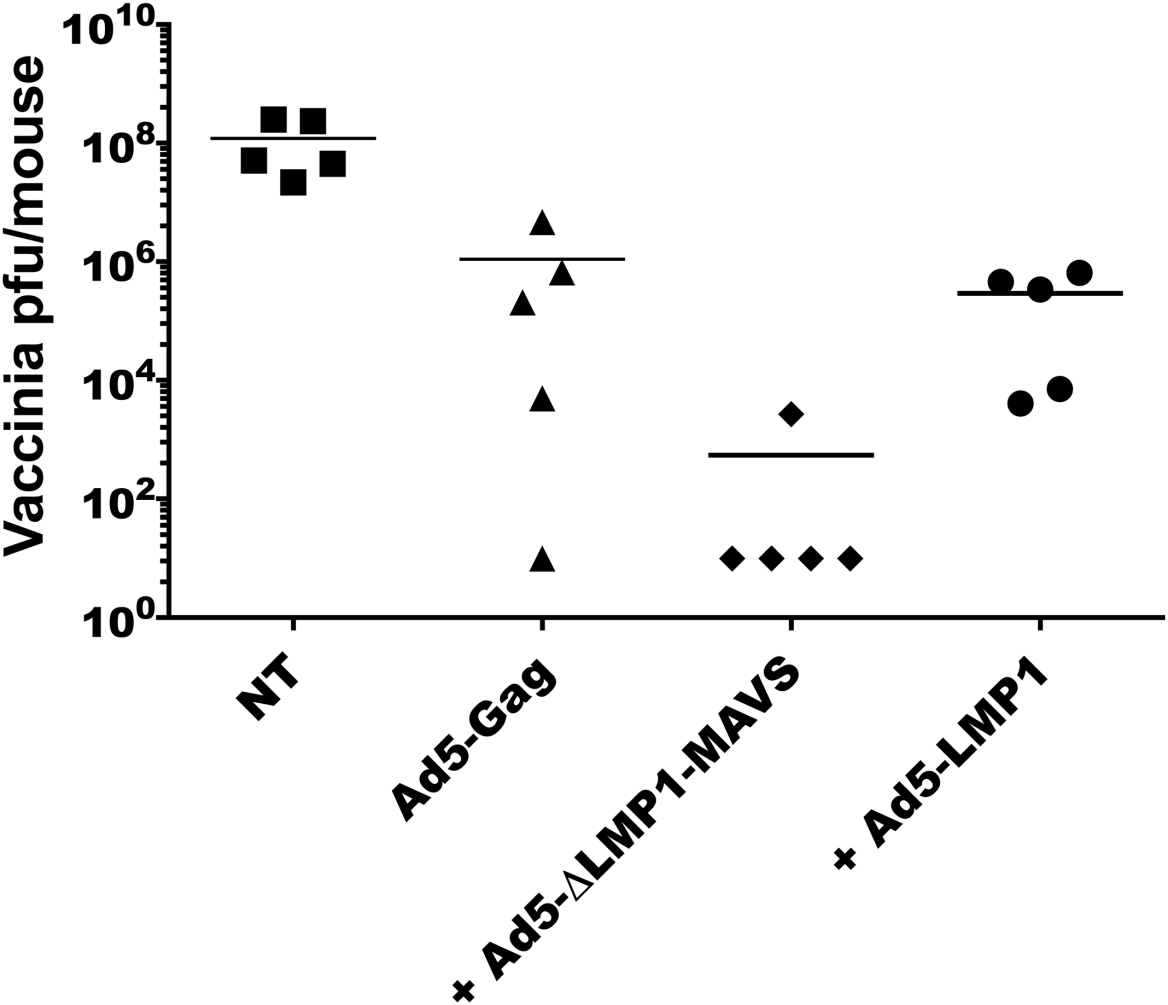
ΔLMP1-MAVS enhances anti-Gag immune responses as an Ad5 viral vector vaccine adjuvant. BALB/c mice were left untreated or vaccinated with a combination of Ad5-Gag and either Ad5-GFP (control), Ad5-ΔLMP1-MAVS, or Ad5-LMP1. Two weeks following vaccination, mice were challenged with vaccinia-gag virus. Vaccinia titers were measured from ovaries after 5 days. NT: no treatment.

Systems biology analysis of the yellow fever vaccine and related studies suggest that type I interferon may contribute to the development of an effective adaptive immune response [51,52]. To evaluate the efficacy of ΔLMP1-MAVS in vaccine model, we vaccinated mice with Ad5 encoding HIV-1 Gag antigen combined with Ad5-GFP (control), Ad5-LMP1, or Ad5-ΔLMP1-MAVS. Mice were vaccinated twice at 2-week intervals. Two weeks following the second vaccination, mice were challenged with 10^7^ PFU of vaccinia-gag virus. As shown in Fig 8, addition of Ad5-ΔLMP1-MAVS resulted in undetectable levels of vaccinia-gag in 4/5 vaccinated animals and a 3-log reduction in mean viral titers compared to Ad5-Gag in the absence of adjuvant. In contrast, the addition of Ad5-LMP1 adjuvant failed to clear virus from vaccinated animals and only modestly reduced mean viral load compared to Ad5-Gag (~0.5-log reduction).

## Discussion

It has long been known that type I interferon inhibits HIV-1 replication, both in vitro and in the clinic [12–14,42,47,53,54]. However, targeted interventions to exploit interferon-mediated viral inhibition, while avoiding toxicity, have so far not been addressed. Of particular interest is the role of innate immune sensors in inducing type I interferon responses. RIG-1 and MDA-5 are sensors for intracellular viral RNA and induce type I interferons [49,55,56]. Both molecules signal through the adapter protein MAVS (IPS-1, VISA), leading to NF-κB induction, type I interferon secretion, and the upregulation of ISGs [23,24,57,58]. During natural HIV-1 infection, RIG-I and MDA-5 have only a limited capacity to respond to HIV-1 RNA [58]. HIV is proposed to avoid RIG-I/MDA-5 sensing through a number of mechanisms, including a recent description of HIV-1 protease-mediated degradation of RIG-I following infection of macrophages [59].

This report evaluated a constitutively active form of MAVS (ΔLMP1-MAVS), and examined the ability of this construct to induce innate immune responses that inhibit HIV-1 viral replication. ΔLMP1-MAVS expression in 293T cells led to high levels of type I interferon expression as measured by reporter assay and RT-PCR analysis. Supernatant from ΔLMP1-MAVS-transfected 293T cells was sufficient to inhibit HIV-1 replication in both TZM-bl cells as well as activated primary human CD4+ T cells. While interferon-mediated HIV-1 inhibition was anticipated based on the literature, the strength of this inhibition was surprising. For example, Wang et al. described RIG-I mediated inhibition of HIV replication in macrophages [60], but did not explore the effect on CD4+ T cells. In addition, the response we observed appears to be independent of NF-κB induction, given that LMP1, a potent inducer of NF-κB, failed to induce more than a modest reduction in viral replication. Consistent with this observation, others have shown only minimal levels of type I interferon induction by full length LMP1 [61].

Viral restriction mediated by ΔLMP1-MAVS appeared to be post-entry and was observed for both HIV and SIV. Supernatant from MAVS-transfected 293T cells inhibited replication of a single cycle SIV at similar levels compared to replication-competent HIV-1 BaL, suggesting that MAVS-induced soluble factor(s) were directly inhibiting virus production within the infected cell, as opposed to inhibition of the spread of virus to new cells within the culture.

Microarray analysis of CD4+ T cell cultured with MAVS-transfected 293T supernatant showed upregulation of a number of interferon stimulated genes, including known and putative HIV-1 restriction factors Viperin, Tetherin, MxB, IFITM1, and ISG20. We also observed upregulation of ISG56/IFIT1, which has been proposed as a putative HIV restriction factor based on evidence of inhibition of HIV protein translation [37]. In addition, OAS2 has also been implicated in detection and inhibition of HIV replication [62], and was upregulated by ΔLMP1-MAVS in both 293T and CD4+ T cells. A number of genes were upregulated only in CD4+ T cells, including the putative HIV restriction factors RTP4 (9-fold upregulation) [63], ZBP1 (16-fold upregulation) [64], and SAMD9L (9-fold upregulation). Finally, realtime PCR analysis showed upregulation of MIP-1β expression, providing an additional potential mechanism of HIV-1 inhibition via MIP-1β binding to CCR5 and CCR5 downregulation [65–67].

The bystander inhibition of HIV-1 replication following ΔLMP1-MAVS transfection suggests that ΔLMP1-MAVS could be used as a therapy, harnessing the HIV inhibitory capacity of type I interferon while avoiding the side effects associated with systemic interferon administration. One approach would be the targeted delivery of ΔLMP1-MAVS to tissue reservoirs of ongoing HIV-1 replication. This could be accomplished by lentiviral vector delivery of ΔLMP1-MAVS. Lentiviral vector pseudotyped with CCR5 tropic HIV-1 Env would be expected to target known HIV-1 reservoirs (lymph node, gut), leading to expression of ΔLMP1-MAVS and induction of type I interferon at tissue sites likely to harbor HIV-1 infected cells. As discussed below, a drug-inducible system could be used to regulate interferon production in transduced cells and prevent interferon desensitization. Future studies are planned to evaluate ΔLMP1-MAVS in the context of influenza A virus infection, also known to be sensitive to type I interferon [68].

In addition, our data have implications for type I interferon and its role in HIV-1 restriction. Previous studies by Goujon and Malim evaluated IFN-α-mediated HIV-1 restriction in human T cells and macrophages [69]. Similar to our findings, they observed that type I interferon does not affect viral entry. In contrast, HIV-1 restriction correlated with inhibition of viral cDNA accumulation, implicating an early post-entry event. In particular, they found that proteasome inhibitors blocked viral inhibition, suggesting a role of ubiquitination. Therefore, our observation of significant upregulation of USP18, involved in de-ubiquitination, would also be consistent with enhanced restriction of HIV-1 by ISG induction.

As has been previously described for full length and recombinant MAVS [30], ΔLMP1-MAVS induced the activation and maturation of dendritic cells and was an effective molecular adjuvant in an HIV-1 Gag vaccine model. ΔLMP1-MAVS adjuvant activity may be related to both direct activation of transduced dendritic cells by Ad5-ΔLMP1-MAVS as well as type I interferon secretion by neighboring transduced cells at the site of injection. Type I interferon has been shown to play an important role in the adaptive immune response. Studies have linked TBK-1 mediated type I interferon induction with the induction of antigen-specific B and T cells in mice in a DNA vaccine model [70]. Type I interferon-receptor mediated signaling was required to induce the adaptive immune response, implicating IFN-α/β secretion in vaccine-mediated protection. As mentioned previously, MAVS induces type I interferon in part via interaction with and aggregation of TBK-1, leading to phosphorylation of IRF-3. Similarly, Kawai et al. showed that MAVS mediated signaling can lead to phosphorylation of both IRF-3 as well as IRF-7 [23]. Importantly, we observed a significant upregulation of IRF-7 in our microarray analysis following trans-activation of CD4+ T cells, suggesting that type I interferon signaling may further enhance IRF-7 mediated IFN-α/β induction. More recently, researchers have explored the mechanisms involved in type I interferon mediated induction of adaptive immunity. Xiao et al. showed that anti-tumor CD8+ T cell responses could be enhanced by direct tumor injection of TLR3/9 agonists, and that this response was dependent on type I interferon [71]. Potential mechanisms include the activation of DC by type I interferon [72]. Systems biology analysis of human responses to yellow fever vaccine induced a type I interferon-mediated response comparable to the MAVS-mediated CD4+ T cell response we present, including upregulation of IRF7 and various ISG including MxB, Viperin, and ISG56 [51]. More recently, Wang et al. showed that TLR7-mediated type I interferon production is critical for the induction of protective CD8+ T cell responses against chronic LCMV [73]. These data further support the role of type I interferon in the induction of adaptive immunity. Studies are ongoing to quantify the cellular and humoral immune responses induced when ΔLMP1-MAVS is used as a vaccine adjuvant. We anticipate that adjuvant responses will be similar to those mediated by STING agonists [74]. One concern regarding the use of constitutive type I interferon inducers such as ΔLMP1-MAVS is potential toxicity. We did not observe overt signs of distress in mice receiving Ad5-ΔLMP1-MAVS compared to controls, despite providing doxycycline to allow constitutive expression throughout the experiment. Further studies will be required to confirm whether vector delivery of ΔLMP1-MAVS avoids toxicity associated with systemic delivery of type I interferons. Similarly, control of ΔLMP1-MAVS expression will be essential to prevent the potential for inflammation associated pathologies. Either short-lived delivery using RNA encoding ΔLMP1-MAVS or tight control of gene expression using a system similar to the Tet-on system evaluated in this study will likely be required for translation to the clinic.

Sandler et al. have studied type I interferon receptor blockade or IFN-α treatment during rhesus macaque SIV infection and disease progression [75]. Paradoxically, both type I interferon receptor blockade with a receptor agonist and treatment with IFN-α led to accelerated disease progression. Of particular interest, Sandler et al. observed a decrease in ISG including OAS2 and MX1 following repeated type I interferon administration, suggesting that pre-challenge exposure to IFN-α led to IFN-desensitization. This has implications for the use of ΔLMP1-MAVS as a HIV-1 therapeutic. A drug inducible system, similar to the doxycycline-inducible AD5-ΔLMP1-MAVS Tet-On vector presented in this study, would allow short bursts of interferon secretion within the tissue by regulated drug administration, avoiding type I interferon induced desensitization of bystander cells. While the Sandler et al. study indicates type I interferon can inhibit adaptive immune responses when given too early in the response, our data suggest that strong type I interferon induction, when coupled to antigen presentation in a viral vector vaccine approach, can enhance immune responses.

The use of a viral vector to deliver ΔLMP1-MAVS provides a number of advantages, including targeting of type I interferon to specific tissues to avoid systemic toxicity, and for prophylactic vaccination the ability to deliver antigen and ΔLMP1-MAVS within the same vector. However, we acknowledge a number of potential disadvantages with ΔLMP1-MAVS, including the possibility that IFN-β is preferentially expressed compared to IFN-α and issues related to the safety of recombinant LMP1 constructs. In conclusion, ΔLMP1-MAVS provides a unique molecule for understanding type I interferon mediated HIV-1 restriction, both as a promising approach for HIV-1 inhibition in a therapeutic setting, and as a novel molecular adjuvant for preventive and therapeutic HIV-1 vaccines.
